# Survival after cessation of immunotherapies in melanoma: A systematic review and meta‐analysis

**DOI:** 10.1111/jdv.20672

**Published:** 2025-04-04

**Authors:** Kristine E. Mayer, Lydia Warburton, Anne Zaremba, Sarah Preis, Yannick Foerster, Tilo Biedermann, Oana‐Diana Persa

**Affiliations:** ^1^ Department of Dermatology and Allergy School of Medicine, Technical University of Munich, Bavarian Cancer Research Center (BZKF) Munich Germany; ^2^ Centre for Precision Health, Edith Cowan University Joondalup Western Australia Australia; ^3^ Department of Medical Oncology Fiona Stanley Hospital Murdoch Western Australia Australia; ^4^ Department of Dermatology, Venerology and Allergology University Hospital Essen Essen Germany; ^5^ Institute for Medical Information Processing, Biometry, and Epidemiology, Pettenkofer School of Public Health LMU Munich Munich Germany

## Abstract

**Background:**

Immune‐checkpoint inhibitor (ICI) therapy elicits durable responses in a subset of patients with advanced melanoma. However, the appropriate timing for treatment cessation remains an unresolved issue. Moreover, some patients are required to discontinue therapy due to the occurrence of severe adverse events. Upon treatment cessation, a subset of patients maintains a durable response, while some patients relapse and require rechallenge with ICI. Criteria for a safe stop of ICI have not been established.

**Objectives:**

The aim of this systematic review and meta‐analysis was to evaluate the durability of response in melanoma patients who discontinued ICI therapy. Furthermore, the outcome of patients who electively stopped therapy was compared to that of patients who discontinued therapy due to adverse events.

**Methods:**

MEDLINE/PubMed, Embase and the Cochrane Library were searched for studies reporting outcomes after ICI discontinuation in patients with advanced melanoma. Pooled 1‐ to 3‐year progression‐free survival (PFS) and overall survival (OS) rates were estimated using random‐effects models. The impact of the reason for treatment discontinuation, therapy regime and treatment duration on relapse‐free survival was evaluated.

**Results:**

Twenty studies including 1832 patients were analysed. The pooled 1‐ and 3‐year PFS rates after therapy stop were 86% (95% CI 80%–91%) and 71% (95% CI 64%–77%). A significantly higher 1‐year PFS rate was observed in patients who electively discontinued treatment in contrast to toxicity‐related therapy cessation (91% vs. 79%). Longer ICI treatment was associated with a higher PFS rate. 1‐ and 3‐year OS rates post ICI treatment discontinuation were 96% (95% CI 91%–99%) and 86% (95% CI 79%–92%).

**Conclusions:**

Most patients remained relapse‐free after ICI treatment. Patients with a treatment duration of at least 2 years are ideal candidates for treatment cessation, while treatment discontinuation may be considered after at least 1 year of ICI.

**PROSPERO number:** CRD42024543781.

## Graphical abstract

This meta‐analysis on immunotherapy discontinuation in metastatic melanoma demonstrated high progression‐free survival rates at 1 year (86%) and 3 years (71%), and high overall survival rates at 1 year (96%) and 3 years (86%). Elective treatment discontinuation and treatment durations exceeding 1 year were associated with improved survival outcomes.

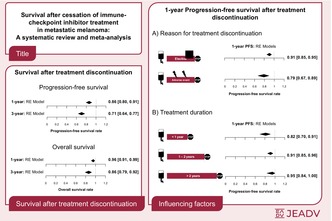

**Why was the study undertaken?**

- To date, prospective clinical trials evaluating the discontinuation of immune checkpoint inhibitor (ICI) therapy in patients with metastatic melanoma are lacking. This systematic review and meta‐analysis were conducted to provide evidence‐based recommendations for clinical practice using currently available data.

**What does this study add?**

- Most melanoma patients who discontinued ICI treatment remained relapse‐free 3 years post‐treatment (71%), suggesting a sustained therapeutic effect. The 3‐year overall survival rate was 86%. Patients who discontinued treatment electively exhibited superior survival outcomes compared to those who discontinued due to adverse events, with 1‐year progression‐free survival (PFS) rates of 91% versus 79%, respectively. Treatment durations exceeding one year were associated with a higher 1‐year PFS after treatment cessation compared to treatments lasting less than 1 year (91% vs. 82%).

**What are the implications of this study for disease understanding and/or clinical care?**

- ICI therapy should be continued for at least 1 year in metastatic melanoma patients to minimize the risk of disease relapse following treatment discontinuation. After a sustained therapeutic response, ICI therapy can be safely stopped. However, regular radiological stagings within the first 3 years post‐treatment discontinuation are essential for early relapse detection, particularly in patients who discontinued treatment due to adverse events.

## INTRODUCTION

Immune‐checkpoint inhibitors (ICI) have significantly prolonged overall survival (OS) in many cancers.^1^, ^2^, ^3^, ^4^ The blockade of cytotoxic T‐lymphocyte‐associated protein 4 (anti‐CTLA‐4) using ipilimumab and programmed cell death protein‐1 (anti‐PD‐1) through nivolumab or pembrolizumab was initially evaluated in patients with advanced melanoma and showed excellent response rates.^1^, ^2^, ^3^ Currently, ICI alongside targeted therapy with BRAF and MEK inhibitors is the standard of care treatment in patients with advanced melanoma. However, the significant improvement in OS comes at the price of immune‐related adverse events (irAE) which can persist even after ICI discontinuation.^5^, ^6^

Interestingly, even in patients presenting with a high tumour burden and cerebral metastasis, the immune response elicited by ICI treatment has the potential to result in complete regression of melanoma in a subset of individuals.^1^, ^7^ Such complete responses may persist beyond treatment discontinuation.^7^, ^8^ However, melanoma can relapse even after initial response, and not all patients respond to re‐challenge with ICI.^9^, ^10^ Conversely, prolonged treatment with immune checkpoint inhibitors (ICIs) is associated with an increased risk of immune‐related adverse events (irAEs). The substantial financial burden of ICI therapy further supports the consideration of treatment cessation upon achieving complete remission (CR). These factors complicate physicians' decisions regarding the discontinuation of effective ICI therapy in the absence of side effects, particularly in the context of a lack of established guidelines for safe ICI cessation. In most clinical trials, patients with advanced melanoma who responded and tolerated the therapy were treated for 2 years.^7^, ^11^ In practice, however, both patients and physicians may be reluctant to electively stop therapy in fear of late tumour recurrence. Conversely, some patients may advocate for an earlier therapy discontinuation due to out‐of‐pocket costs and desire for normalcy.

Another situation in which physicians are forced to stop ICI treatment is cases of Grade III and IV irAE.^12^ Most irAE are associated with better response and a positive effect on survival.^13^ However, especially when severe irAE occur in the early phase of treatment, the decision to stop or reinitiate ICI therapy after resolution of the irAE is challenging. To answer the question of whether early elective ICI treatment discontinuation in patients with complete or partial response upon ipilimumab/nivolumab is safe, a prospective, single‐arm intervention study has been initiated (NCT05652673).^14^

Current data on long‐term outcomes after discontinuation of ICI treatment from melanoma patients are mostly derived from retrospective analyses with only a few clinical trials. Only 10.1% of patients with advanced melanoma in CR recruited in the Keynote 001 experienced a relapse after cessation of a 2‐year treatment with pembrolizumab.^7^ In line with these findings, retrospective studies showed that patients with a CR are less likely to relapse upon ICI cessation compared to patients with partial remission (PR) or stable disease (SD).^15^, ^16^

This systematic review and meta‐analysis aims to summarize the currently available survival data on ICI cessation in patients with irresectable Stage III or Stage IV melanoma. It investigates differences in progression‐free survival (PFS) between patients who electively discontinued treatment and those who stopped due to irAE. Furthermore, the effect of mono‐ and combination therapy, treatment duration, as well as response category on survival outcome was evaluated.

## METHODS

### Search strategy and study selection

This systematic review and meta‐analysis was registered on PROSPERO (CRD42024543781). The Preferred Reporting Items for Systematic Reviews and Meta‐Analyses (PRISMA) statement was used as a guidance.^17^ MEDLINE/PubMed, Embase (via Ovid) and the Cochrane Central Register of Controlled Trials (via Ovid) were searched from inception to 15 May 2024, using the following input: (immunotherapy OR “immune checkpoint” OR nivolumab OR ipilimumab OR pembrolizumab) AND (“melanoma”) AND (metastatic OR advanced) AND (stop OR stopped OR discontinuation OR discontinue OR withdrawal OR withdrawn OR treatment free). References and abstracts were imported into Endnote. Duplicates were removed with the respective Endnote function and manually. KEM and ODP independently reviewed titles, abstracts and full‐text manuscripts for study eligibility. Discrepancies were solved by discussion.

Publications in other languages than English and German, case reports, reviews, comments, editorials, guidelines, conference abstracts, cell culture and animal studies, as well as studies with treatment of an anti‐CTLA‐4 antibody only were excluded. For inclusion, the studies had to (1) involve at least 20 metastatic melanoma patients ≥18 years old under ICI treatment and (2) report overall or patient‐level PFS and/or OS after immunotherapy discontinuation for at least a subset of patients. PFS and OS were defined as the period from therapy discontinuation until progression or censoring and death or censoring, respectively.

### Quality assessment

KEM and ODP independently evaluated the risk of bias of each study using a modified, previously described Newcastle–Ottawa Scale Score^18^, ^19^: (1) cohort representative of advanced melanoma patients; (2) ICI treatment documented in medical records; (3) outcome of interest shown to be absent at the start of the study; (4) outcomes assessed using objective, predefined criteria; (5) adequate follow‐up duration for outcomes to occur (≥12 months); and (6) adequate cohort follow‐up (<10% lost to follow‐up). Higher quality was assumed if a study met at least four of the above‐mentioned criteria.^19^ Again, discrepancies between the authors were resolved by discussion.

### Data collection

One‐year, 2‐year and 3‐year PFS as well as OS data were extracted from the studies meeting the inclusion criteria. If the data were not explicitly mentioned, corresponding authors were contacted and asked for the precise values. Otherwise, PFS and OS rates were determined from published Kaplan‐Meier curves or swimmer plots using WebPlotDigitizer v4.8. Data were collected for the following subgroups, if applicable: (1) elective treatment discontinuation and therapy stop due to irAE; (2) ICI treatment with anti‐PD‐1 monotherapy versus the anti‐PD‐1/anti‐CTLA‐4 combination therapy; (3) different treatment duration with ICI before therapy discontinuation; (4) best overall response; and (5) time to response. The treatment duration of ICI was classified as up to 1 year, 1–2 years and more than 2 years on a patient level according to given swimmer plots. Kaplan‐Meier curves of the respective subgroups were drawn in GraphPad Prism v10.4.0, and survival data were extracted.

### Data synthesis and analysis

The meta^20^ and metafor^21^ packages in RStudio v2024.04.2 + 764 were used for statistical analyses. After calculating the respective patient proportions for each study and outcome measure, the variance‐stabilizing Freeman–Tukey double‐arcsine transformation was applied.^22^ To account for heterogeneity between studies, a random‐effects model, namely the restricted maximum‐likelihood approach, based on the inverse‐variance method, was applied.

Cochran's Q test and the *I* ^2^ statistic were used to determine heterogeneity between study cohorts. Leave‐one‐out sensitivity analysis, funnel plots and Egger's regression test helped to identify influential studies and evaluate publication bias.^21^

Differences between the above‐mentioned subgroups were assessed by chi‐square tests. *p*‐values <0.05 were considered statistically significant. ChatGPT was used for language editing of the abstract, introduction and discussion part of this manuscript.

## RESULTS

### Search results and study characteristics

**FIGURE 1:**
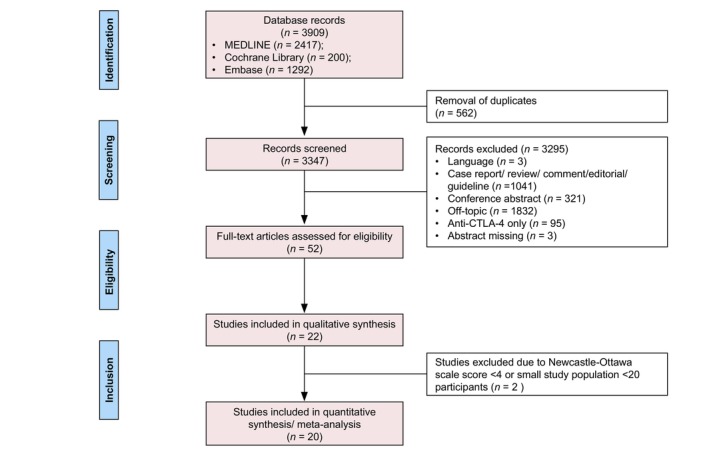
Preferred Reporting Items for Systematic Reviews and Meta‐Analyses flow diagram for study identification and selection.

The database search revealed 3909 records including 562 duplicates. By screening titles and abstracts, 3295 records were excluded due to publication type, language, or different topic. Fifty‐two full‐text articles were assessed for eligibility. Thirty articles were excluded as they were off‐topic or did not contain the relevant data. Another two publications were excluded because of a Newcastle–Ottawa Scale Score < 4 or study population < 20 participants. Finally, 20 studies were included in the meta‐analysis (Figure 1).^7^, ^15^, ^16^, ^23^, ^24^, ^25^, ^26^, ^27^, ^28^, ^29^, ^30^, ^31^, ^32^, ^33^, ^34^, ^35^, ^36^, ^37^, ^38^, ^39^

All studies were of high methodological quality, see the modified Newcastle–Ottawa Scale Score (Table 1). In total, 1896 ICI‐treated metastatic melanoma patients were included. 18 studies included patients after elective treatment stop and 13 studies included patients with toxicity‐related discontinuation. Nine studies reported patients with anti‐PD‐1 monotherapy only, 2 studies with combination therapy only and the rest included patients with anti‐PD‐1 monotherapy or combination therapy. Median treatment duration was lower for studies or study subgroups with patients who discontinued treatment due to toxicities compared to elective treatment discontinuation (Table 1).

**TABLE 1:** Characteristics of included studies.

Study	*N*	Reason for treatment discontinuation	Type of treatment	Median treatment duration (months)	Newcastle–Ottawa scale score
Asher et al. 2021^23^	106	Elective and toxicity	Ipi/Nivo, Nivo, Pembro	15.2	5
Dimitriou et al. 2021^24^	125	Elective and toxicity	Ipi/Nivo, Ipi/Pembro, Nivo, Pembro	22/3	5
Ellebaek et al. 2023^25^	140	Elective and toxicity	Ipi/Nivo, anti‐PD‐1, anti‐PD‐1 + experimental	7.8 (10.3/3.9)	5
Ferdinandus et al. 2022^26^	38	Elective and toxicity	Ipi/Nivo, Nivo, Pembro	24	6
Gibney et al. 2021^27^	52	Elective and toxicity	Ipi/Nivo, Ipi/Pembro, Nivo, Pembro	12/4	5
Jansen et al. 2019^16^	185	Elective	Nivo, Pembro	12	5
Kartolo et al. 2023^28^	39	Elective and toxicity	Anti‐PD‐1, anti‐PD‐1/anti‐CTLA‐4	24/6	5
Ladwa et al. 2017^29^	29	Elective	Nivo, Pembro	12.5/9	6
Ochenduszko et al. 2023^30^	35	Elective	Nivo, Pembro	23.4	5
Perez et al. 2022^31^	46	Elective	Ipi/Nivo, Nivo, Pembro	9.6	5
Persa et al. 2021^15^	87	Elective	Anti‐PD‐1, anti‐PD‐1/anti‐CTLA‐4	15	5
Pokorny et al. 2021^32^	52	Elective	Nivo, Pembro	11.1	6
Robert et al. 2018^7^	89	Elective and toxicity	Pembro	23/6	5
Rubatto et al. 2023^33^	237	Elective and toxicity	Nivo, Pembro	32.4/19	5
Schadendorf et al. 2017^34^	98	Toxicity	Ipi/Nivo	1.4	5
Schank et al. 2021^35^	45	Elective and toxicity	Ipi/Nivo, anti‐PD‐1, ipilimumab	21	5
Valentin et al. 2019^36^	65	Elective	Anti‐PD‐1	23	4
Warburton et al. 2020^37^	70	Elective and toxicity	Pembro	11.8	6
Warburton et al. 2023^38^	34	Toxicity	Ipi/Nivo	2.1	6
Zeijl et al. 2021^39^	324	Elective and toxicity	Anti‐PD‐1	7–12	5

*Note*: The number of patients, the reason for treatment discontinuation, the type of treatment, the median treatment duration and the Newcastle–Ottawa Scale Score of the respective studies are shown. If available, the median treatment duration for the whole study cohort as well as the subgroups elective/toxicity is depicted.

### Overall PFS and OS rates after ICI treatment discontinuation

**FIGURE 2:**
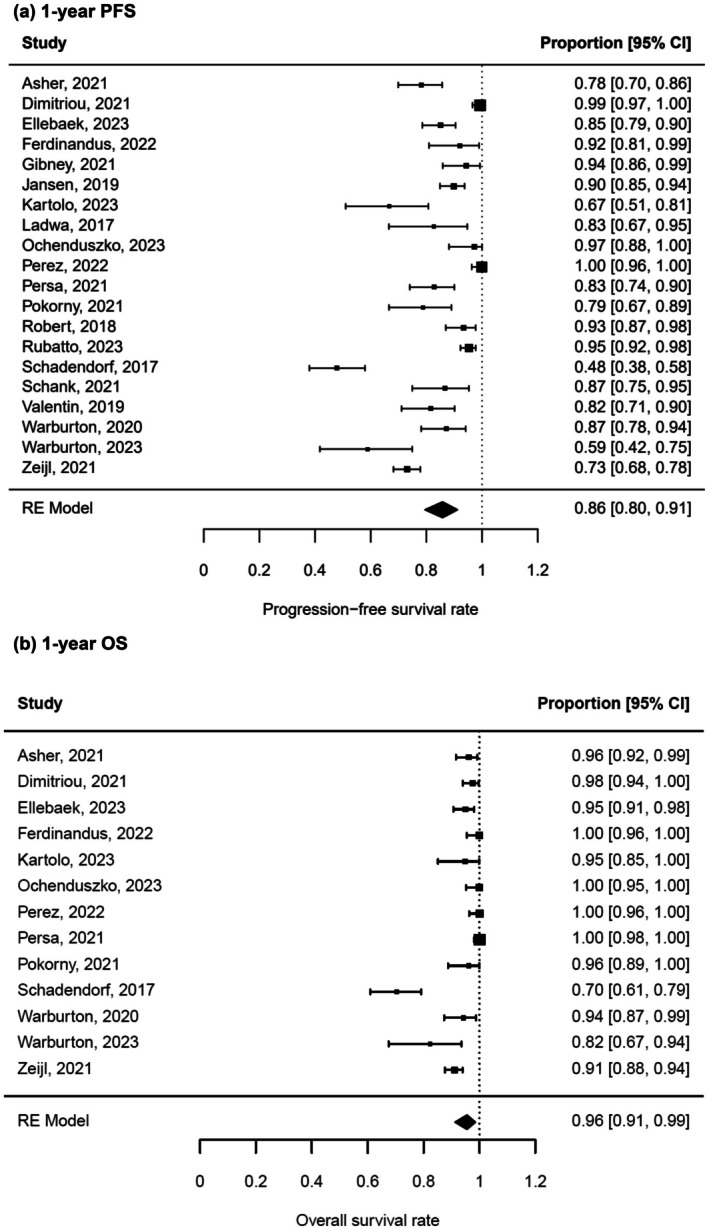
One‐year progression‐free survival (PFS) and 1‐year overall survival (OS) after treatment discontinuation. Random‐effects (RE) meta‐analysis of 1‐year PFS rate (a) and 1‐year OS rate (b) in metastatic melanoma patients treated with immune‐checkpoint inhibitors (ICI). Survival rates are depicted as proportions with 95% confidence intervals (CI).

The weighted mean 1‐year PFS rate for all metastatic melanoma patients after therapy discontinuation was 86% (95% CI 80%–91%) (Figure 2a). Large heterogeneity was present between cohorts (*I* ^2^ = 92%, *p* < 0.0001). The 1‐year OS rate was even higher at 96% (95% CI 91%–99%) (Figure 2b). Again, large heterogeneity was detected (*I* ^2^ = 87%, *p* < 0.0001). Leave‐one‐out sensitivity analysis showed that the study Schadendorf et al. 2017^34^ reported distinctly lower PFS and OS compared to the other studies. Visual inspection of the funnel plots and Egger's regression test did not indicate a publication bias (Figure 1a,b).

**FIGURE 3:**
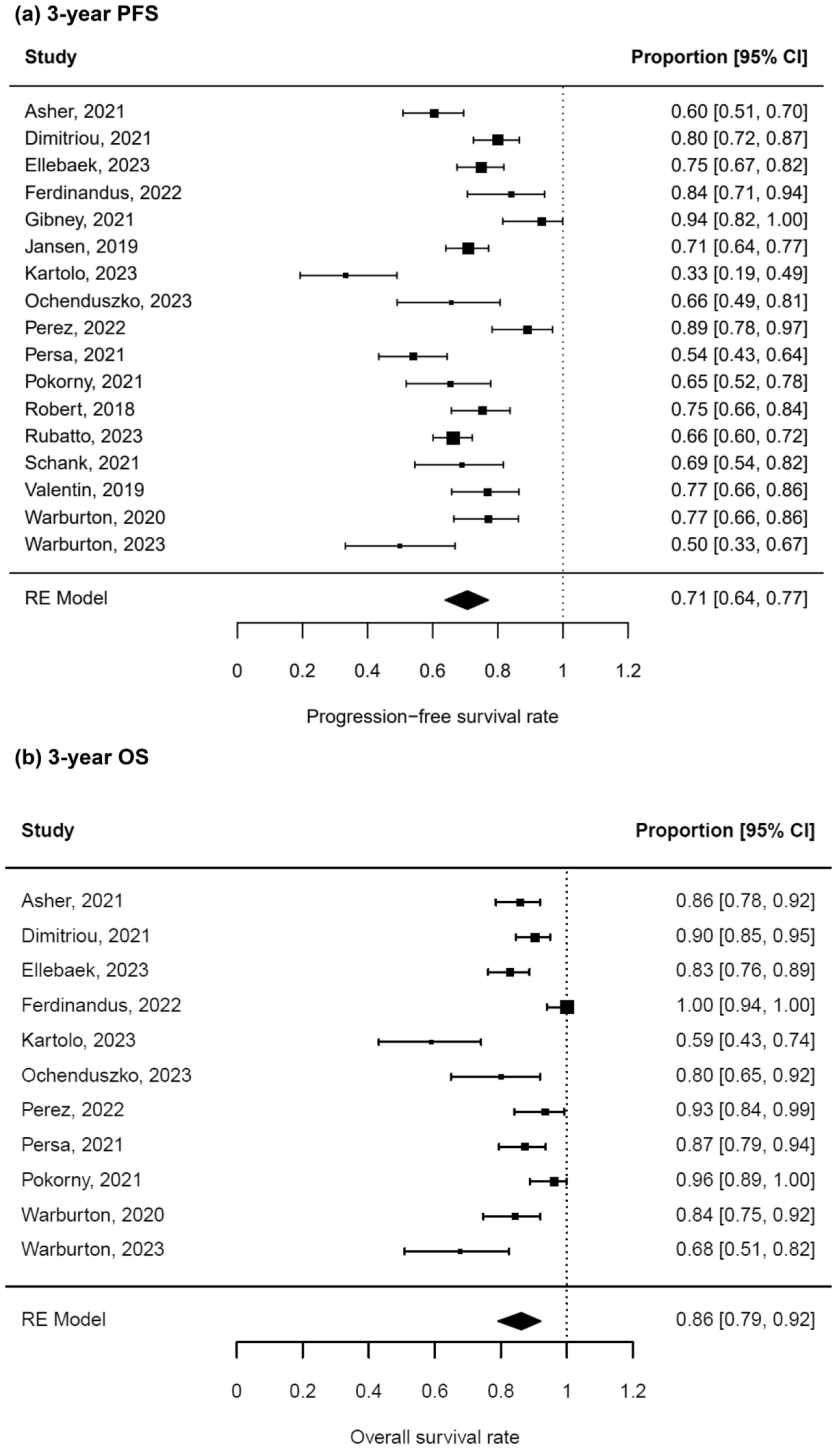
Three‐year PFS and 3‐year OS after treatment discontinuation. RE meta‐analysis of 3‐year PFS rate (a) and 3‐year OS rate (b) in metastatic melanoma patients after ICI treatment. Survival rates are depicted as proportions with 95% CI.

Three‐year PFS rate after ICI treatment was 71% (95% CI 64%–77%) (Figure 3a) and 3‐year OS rate was 86% (CI 79%–92%) (Figure 3a). Again, heterogeneity of the included studies was high (*I* ^2^ = 86% and 84%) and funnel plot analysis did not show asymmetry (Figure 1c,d). As expected, 2‐year PFS and OS rates ranged between the above‐mentioned survival data of 1 and 3 years, namely at 74% (95% CI 66%–81%) and 88% (95% CI 81%–94%), respectively.

### PFS after elective and toxicity‐related discontinuation

**FIGURE 4:**
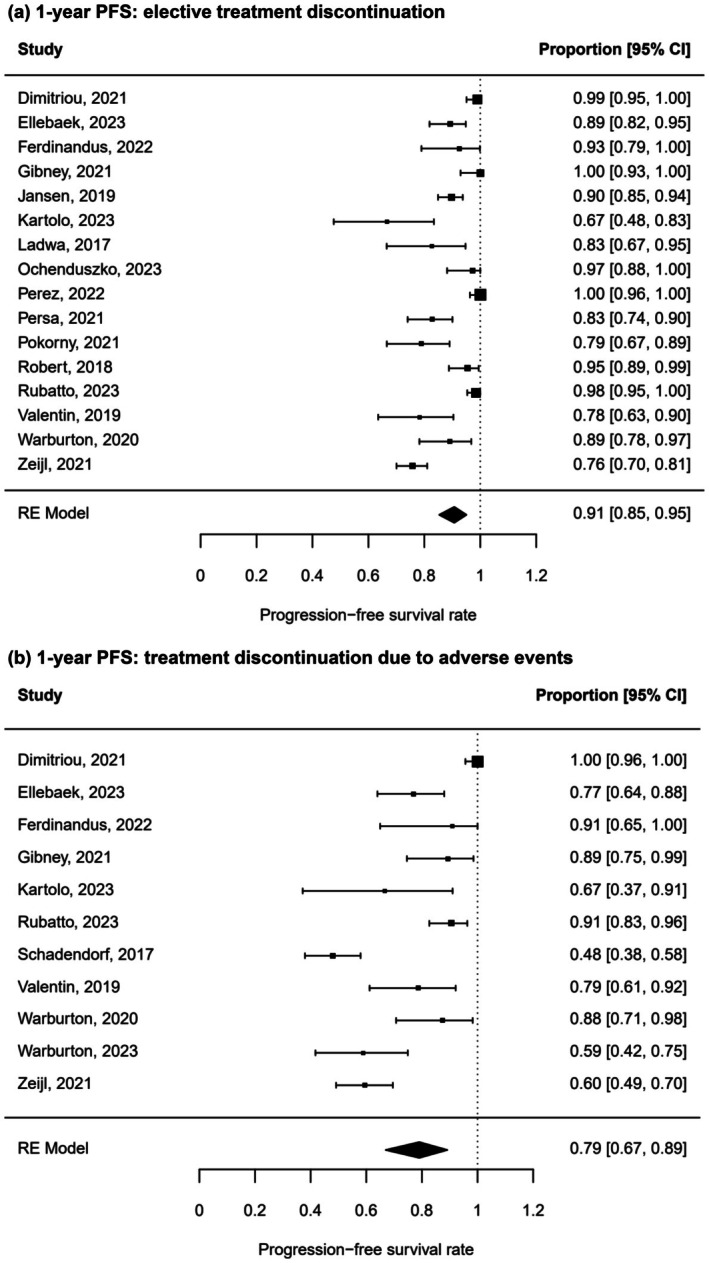
One‐year PFS stratified by reason of treatment discontinuation. RE meta‐analysis of 1‐year PFS rate in metastatic melanoma patients after ICI treatment who discontinued electively after response in accordance with their physicians (a) or due to severe adverse events (b).

Next, available data were stratified by reason for treatment discontinuation. In total, 1202 patients stopped treatment electively in agreement with their doctor, and 483 patients had to discontinue treatment because of irAE. The pooled 1‐year PFS rate after elective treatment discontinuation was 91% (95% CI 85% to 95%) (Figure 4a). For patients who stopped ICI treatment due to toxicity, however, the 1‐year PFS rate was significantly lower (chi‐square test: *p* < 0.0001) with only 79% (95% CI 67%–89%) (Figure 4b). The extent of heterogeneity within the subgroups was comparable to the overall cohort (*I* ^2^ = 86% and 88% for elective and toxicity‐related treatment discontinuation respectively). Leave‐one‐out sensitivity analysis revealed a considerable impact of Dimitriou et al. 2021 on the 1‐year PFS rate of toxicity‐related treatment discontinuation. Again, funnel plot analysis and Egger's regression test did not show any indication of a publication bias.

### PFS after treatment with anti‐PD‐1 monotherapy and combination therapy

**FIGURE 5:**
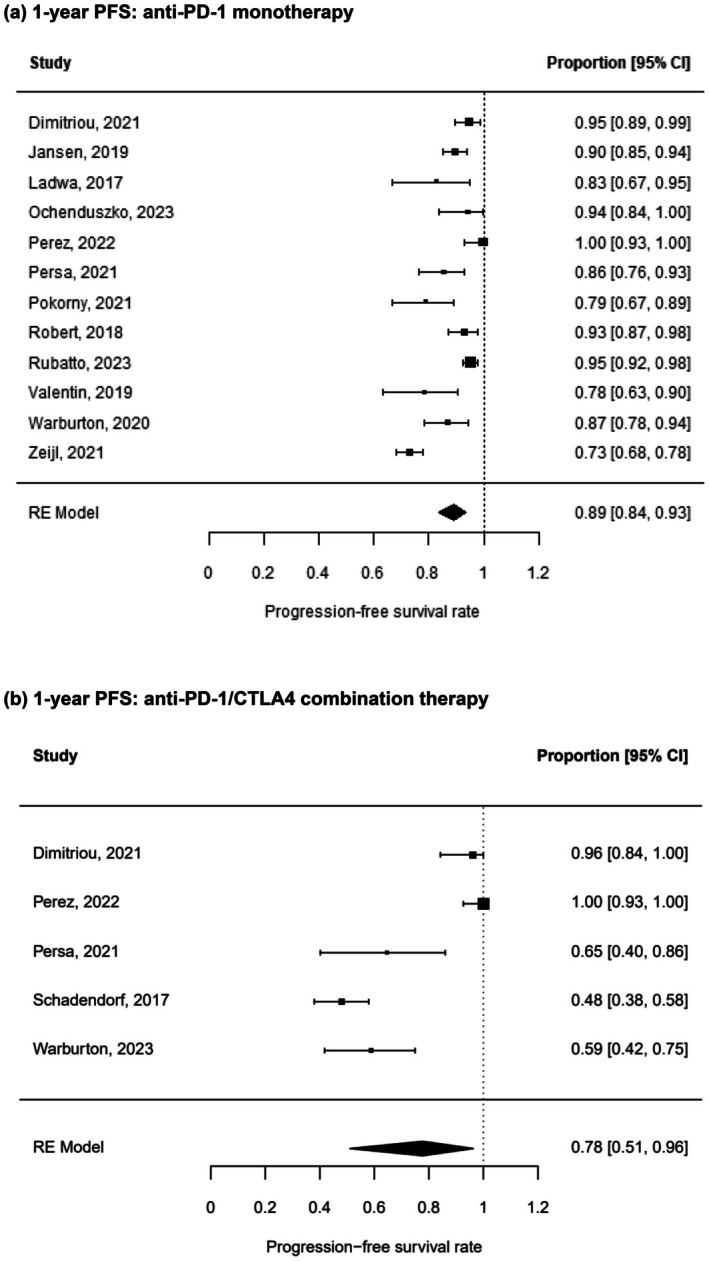
One‐year PFS stratified by treatment regimen. RE meta‐analysis of 1‐year PFS rate in metastatic melanoma patients after ICI treatment who were treated either with anti‐programmed death 1 (PD‐1) monotherapy (a) or with anti‐PD‐1/anti‐cytotoxic T‐lymphocyte‐associated protein 4 (CTLA‐4) combination therapy (b).

To evaluate if the therapeutic regime has an influence on survival after treatment discontinuation, data from patients who received anti‐PD‐1 therapy only and from patients who were treated with a combination therapy including an anti‐CTLA‐4 antibody were analysed. One year post treatment, 89% (95% CI 84%–93%) of the patients under anti‐PD‐1 monotherapy had an ongoing response (Figure 5a). Of the patients on anti‐PD‐1/anti‐CTLA‐4 combination therapy, only 78% (95% CI 51%–96%) were relapse‐free 1 year off‐treatment (Figure 5b). Chi‐square test could identify an influence of treatment modality on PFS (*p* < 0.0001). Leave‐one‐out sensitivity analysis did not reveal any outlying cohorts. Again, there was no indication of a publication bias.

### Influence of ICI treatment duration on PFS off‐treatment

**FIGURE 6:**
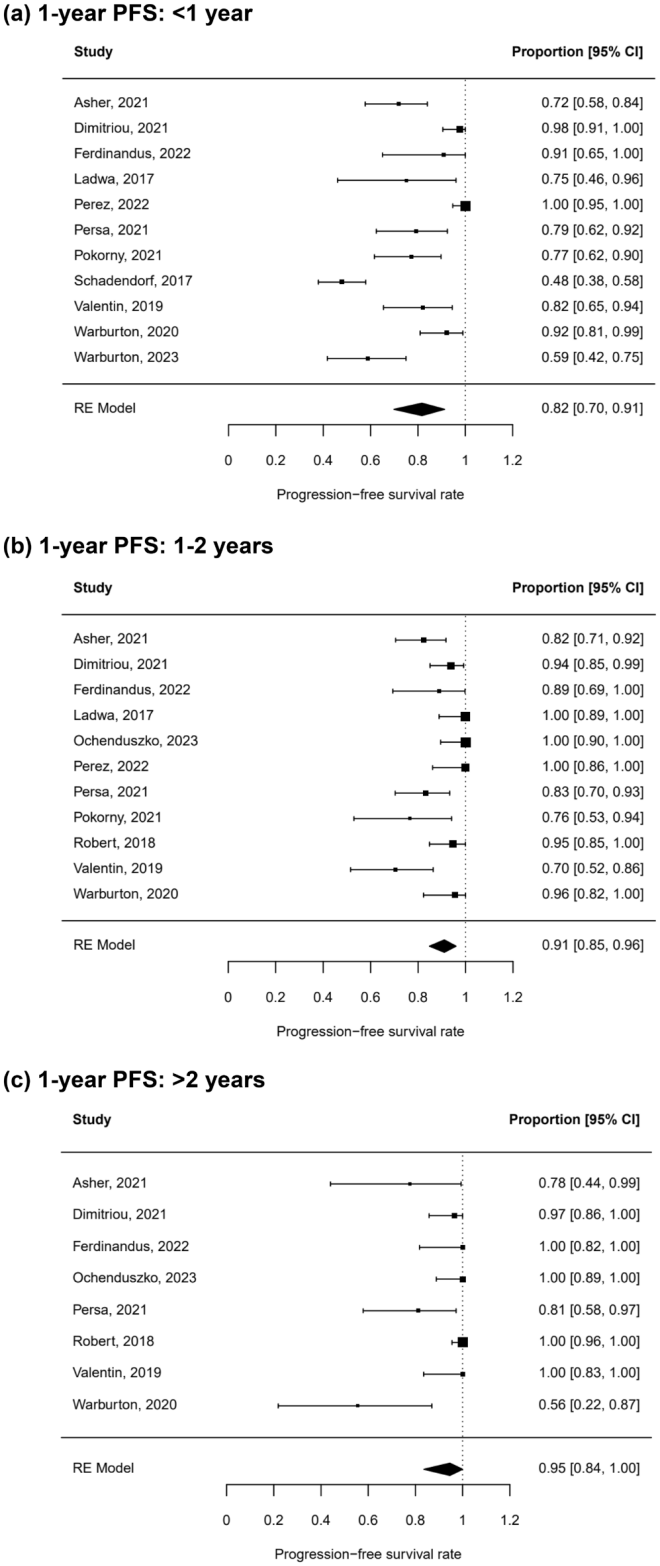
One‐year PFS stratified by treatment duration. RE meta‐analysis of 1‐year PFS rate in metastatic melanoma patients after ICI treatment who were treated for less than 1 year (a), 1–2 years (b) or more than 2 years (c).

Next, the impact of the duration of ICI on PFS after treatment discontinuation should be deciphered. Treatment duration was stratified into up to 1 year, 1–2 years and more than 2 years. Of note, in most clinical trials, treatment was stopped after 2 years. 405, 308 and 135 patients could be included in the respective groups. If there were less than 5 patients per study and subgroup, the study was excluded from the analysis. In patients treated for less than 1 year, the 1‐year PFS rate off‐treatment was 82% (95% CI 70%–91%) (Figure 6a). Patients treated for 1–2 and more than 2 years both had higher PFS rates with 91% (95% CI 85%–96%) and 95% (95% CI 84%–100%) revealing an influence of treatment duration on PFS after therapy stop (Figure 6b,c, chi‐square test: *p* < 0.0001). Leave‐one‐out sensitivity analysis did not reveal any outlying cohorts. Again, there was no indication of a publication bias.

### PFS after treatment according to response category

**FIGURE 7:**
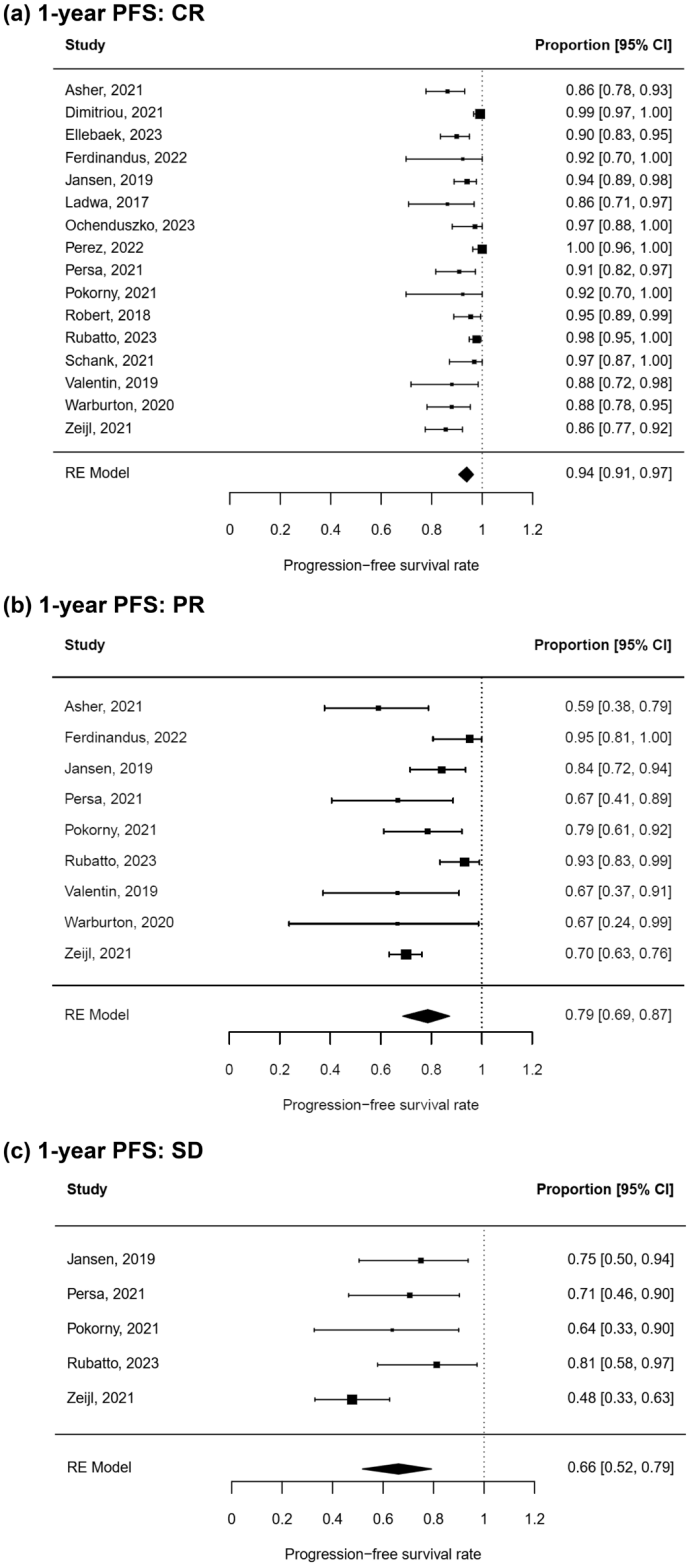
One‐year PFS stratified by response category. RE meta‐analysis of 1‐year PFS rate in metastatic melanoma patients after ICI treatment with complete response (CR) (a), with partial response (PR) (b) or with stable disease (SD) (c).

As expected, the best overall response has a major impact on post treatment PFS. Patients who achieved a CR had a significantly higher 1‐year PFS rate post treatment with 94% (95% CI 91%–97%) (Figure 7a) than patients with a PR or SD with 79% (95% CI 69%–87%) and 66% (95% CI 52%–79%), respectively (Figure 7b,c, chi‐square test: *p* < 0.0001). Time to response using a cut‐off of 6 months did not significantly influence the 1‐year PFS rate off treatment (Figure 2a,b; chi‐square test: *p* = 0.67). For all analyses, leave‐one‐out sensitivity analysis did not reveal any outlying cohorts, and the funnel plots and Egger's regression tests indicated no publication bias.

## DISCUSSION

This meta‐analysis summarizes PFS and OS data after ICI discontinuation in advanced melanoma revealing three key findings. First, PFS and OS rates up to three years after therapy stop were sustainably high (Figure 2; Figure 3). Second, patients under elective treatment discontinuation exhibit better 1‐year PFS (91%) in comparison to toxicity‐related therapy stop (79%) (Figure 4). Finally, longer treatment duration of ICI treatment is associated with a higher 1‐year PFS after treatment discontinuation (Figure 6).

ICI treatment has transformed the therapeutic landscape for metastatic melanoma. Unlike conventional therapies, ICIs have the potential for eliciting durable responses.^11^ Consequently, treatment discontinuation after achieving a durable response may be reasonable despite possible relapse.^40^ While in trials with ipilimumab, patients received only four doses, some of the anti‐PD‐1 inhibitors trials did not have an upper limit of treatment duration but allowed stopping therapy after 2 years.^11^, ^41^ Asher and colleagues propose a cut‐off of at least 18 months for elective treatment discontinuation,^23^ while Jansen and colleagues showed that treatment durations of less than 6 months were significantly associated with relapse.^16^ To date, the necessary treatment duration to achieve and sustain a response is still unclear. Our analysis showed that the longer the ICI treatment lasted, the higher the 1‐year PFS rate was after therapy stop. Patients treated for more than two years had an impressive 1‐year PFS rate of 95%, while the rate was 91% for patients treated for 1–2 years and 82% for patients with treatment less than 1 year. Even though some time‐dependent bias must be assumed, this is in line with studies in other tumour entities reporting a higher risk of relapse for shorter treatment duration.^42^, ^43^ Analysis of the MELBASE cohort indicates that prolonged treatment beyond 2 years does not significantly improve survival and proposes 1 year as cut‐off for ICI treatment (Asher et al., ASCO 2024). Overall, further randomized clinical trials are required to shed light on when to electively stop an immunotherapy after response.^14^, ^44^ When deciding to stop treatment, careful consideration of the best overall response is important. In our analysis, patients with CR had significantly better 1‐year PFS following treatment (94%) compared to those with PR or SD (79% and 66%, respectively; Figure 7). In contrast, a longer time to response was not associated with poorer survival after treatment cessation (Figure S2). However, the quality of this analysis is limited by the small sample size per group. In addition to clinical parameters, biomarkers and imaging techniques may help select eligible patients and determine a suitable time point to discontinue treatment. Some of the studies included in this meta‐analysis identified parameters associated with improved survival post‐treatment discontinuation. Patients without detectable circulating tumour DNA (ctDNA) exhibited a lower risk for recurrence compared to patients with persistent ctDNA at the time of treatment cessation.^37^, ^38^ Moreover, complete metabolic responses confirmed by FDG‐PET prior to treatment discontinuation have been shown to correlate with improved survival.^25^, ^26^, ^35^ Taking this into account, results from liquid biopsy, imaging tools and further clinical parameters may be combined to identify patients suitable for (early) treatment discontinuation.

Our findings support elective cessation of ICI therapy in the clinical routine. Irrespective of the reason for treatment cessation, the risk for relapse was low, with a 3‐year PFS rate of 71% and a 3‐year OS rate of 86%, indicating durable effects post‐treatment. Over the 3 years, a decline in PFS and non‐melanoma‐specific OS rates was observed. Secondary resistance or an insufficiently lasting T‐cell response may explain the recurrence of the tumour in some cases. Accordingly, regular stagings in the first 3 years after treatment discontinuation are reasonable. Interestingly, a large proportion of patients with relapse upon treatment discontinuation seem to respond to re‐challenge with ICI, further supporting the rationale for cessation of ICI.^15^, ^16^ However, as the comparison with a respective control group who continued ICI treatment is missing, it is impossible to determine the actual effect of treatment discontinuation. The results of the ongoing Safe Stop Ipi‐Nivo will hopefully provide further evidence that ICI can safely be stopped in the context of complete or partial response.^14^

Our meta‐analysis also indicates a significant difference in PFS depending on the reason for treatment discontinuation, namely elective or due to irAE (Figure 4). The 1‐year PFS rate was distinctly higher in patients who stopped treatment electively (91%) in comparison with patients who had to discontinue therapy because of toxicity (79%). One reason for this observed difference could be the proportion of complete and partial responses. Patients with a best overall response of PR and SD have a higher risk for relapse compared to patients with a CR.^39^ Commonly, patients who electively stop ICI are more likely to be in CR, whereas in patients discontinuing treatment due to toxicity, response assessment may be pending at treatment cessation.^34^, ^36^, ^38^ The reduced PFS rate observed in the toxicity group may be attributable to a shorter treatment duration, which could reflect a less robust immune response, or to the management of irAE with immunosuppressive agents attenuating antitumoral responses.^45^

The present analysis reveals a notable difference in PFS between patients receiving anti‐PD‐1 monotherapy and those undergoing anti‐PD‐1/anti‐CTLA‐4 combination therapy (Figure 5). The significantly higher 1‐year PFS rate observed in monotherapy‐treated patients may be attributed to clinical differences between the subgroups, although this hypothesis cannot be conclusively established due to a lack of patient‐level data. It is plausible that patients on combination therapy presented with a higher tumour burden, a greater proportion of brain metastases, and additional risk factors associated with poorer prognosis. Notably, some studies included in this analysis reported a higher incidence of treatment discontinuation due to toxicity among patients receiving combination therapy, which could adversely impact outcomes.^34^, ^38^ As the reasons for therapy discontinuation are linked to survival, the findings may be significantly influenced by this factor.

This meta‐analysis has several limitations. As only two randomized controlled trials reported survival outcomes for patients who discontinued ICI separately,^7^, ^34^ mainly retrospective studies were included. Some studies had to be excluded as the reported survival data also contained data from patients who discontinued treatment due to progressive disease^46^, ^47^ or the melanoma patients could not be identified from a basket trial.^8^ Data were mainly extracted from published survival curves and swimmer plots with a web tool. Therefore, data extraction accuracy was limited by figure resolution. If data were not accessible, the studies were excluded from respective analyses. Importantly, significant heterogeneity in many of the analyses conducted was observed. We suspect that this is related to the fact that most studies reported retrospective real‐world data with rather heterogeneous study cohorts. For example, some studies on elective treatment discontinuation only included patients with CR, while in others also patients with PR and SD were also included.

Nevertheless, the analysis has key implications for clinical care. It demonstrates that ICI treatment response can continue beyond therapy stop. It shows that longer treatment duration is associated with a higher rate of PFS. Accordingly, patients with a treatment duration of at least 2 years are considered optimal candidates for treatment cessation, whereas discontinuation of treatment may be considered after a minimum of 1 year of ICI therapy. Nevertheless, further trials are needed to answer the question of whether shorter treatment with responders is equally effective. As long as reliable biomarkers for toxicity development as well as durable response are not available, the decision regarding the timing of ICI cessation and selection of patients for treatment stop will continue to pose a challenge in daily practice.

## CONCLUSIONS

Most patients with metastatic melanoma who electively discontinued their ICI treatment after response remained relapse‐free. Even toxicity‐related treatment discontinuation was associated with a relatively high 1‐year PFS rate. Longer treatment duration seems to have a beneficial effect on avoiding relapses. Prospective clinical trials and biomarkers are needed to select patients suitable for stopping immunotherapy without compromising clinical outcomes.

## AUTHOR CONTRIBUTIONS

**Kristine E. Mayer**: conceptualization, data acquisition, methodology, writing—original draft, visualization; **Lydia Warburton**: data acquisition, writing—review and editing; **Anne Zaremba**: data acquisition; **Sarah Preis:** methodology; **Yannick Foerster**: writing—review and editing; **Tilo Biedermann**: writing—review and editing. **Oana‐Diana Persa:** conceptualization, data acquisition, writing—review and editing.

## FUNDING INFORMATION

None.

## CONFLICT OF INTEREST STATEMENT

KEM received travel support from Novartis and is a member of the advisory board of Imnotech GmbH, which holds patents on an anti‐CD2 and an anti‐CD7 single‐domain antibody. LW holds FHRI Fund and Raine Foundation Clinician Research Fellowship, received personal honoraria from Bristol Myers Squibb, received travel support from Merck Sharp & Dohme and is member of the advisory board of Merck Sharp & Dohme, Novartis and Bristol Myers Squibb, AZ received a grant from the Hiege Stiftung and travel support from Novartis, Sanofi Genzyme and Bristol‐Myers Squibb. SP received speaker's honoraria from Janssen, Novartis and Abbvie. YF received travel support from Merck Sharp & Dohme. TB received grants from Almirall, Celgene‐BMS, Lilly, Novartis, Sanofi‐Genzyme, Regeneron and Viatris, received consulting fees from AbbVie, Alk‐Abello, Almirall, Boehringer‐Ingelheim, Leo Pharma, Lilly, Novartis, Sanofi‐Genzyme and Viatris, received honoraria from Alk‐Abello, Almirall, GSK, Leo Pharma, Lilly, Novartis, Sanofi‐Genzyme, Regeneron, and is a member of the advisory board of Alk‐Abello, Almirall, Boehringer‐Ingelheim, Leo Pharma, Lilly, Novartis, Sanofi‐Genzyme and Viatris. ODP received personal honoraria from Merck Sharp & Dohme and Almirall, received travel support from Kyowa Kirin, Pierre Fabre, Sanofi and Sun Pharma and is member of the advisory board of Bristol Myers Squibb and Sanofi.

## ETHICAL APPROVAL

For this systematic review and meta‐analysis, no ethical approval was needed.

## Supporting information


Figure S1.

Figure S2.

## Data Availability

The data that support the findings of this study are available from the corresponding author upon reasonable request.
