# Supplementary figures and images for: Survival after cessation of immunotherapies in melanoma: A systematic review and meta‐analysis
Source: J Eur Acad Dermatol Venereol. 2025 Apr 4;39(11):1961–74. doi: 10.1111/jdv.20672 (PMC12553132; doi:10.1111/jdv.20672)

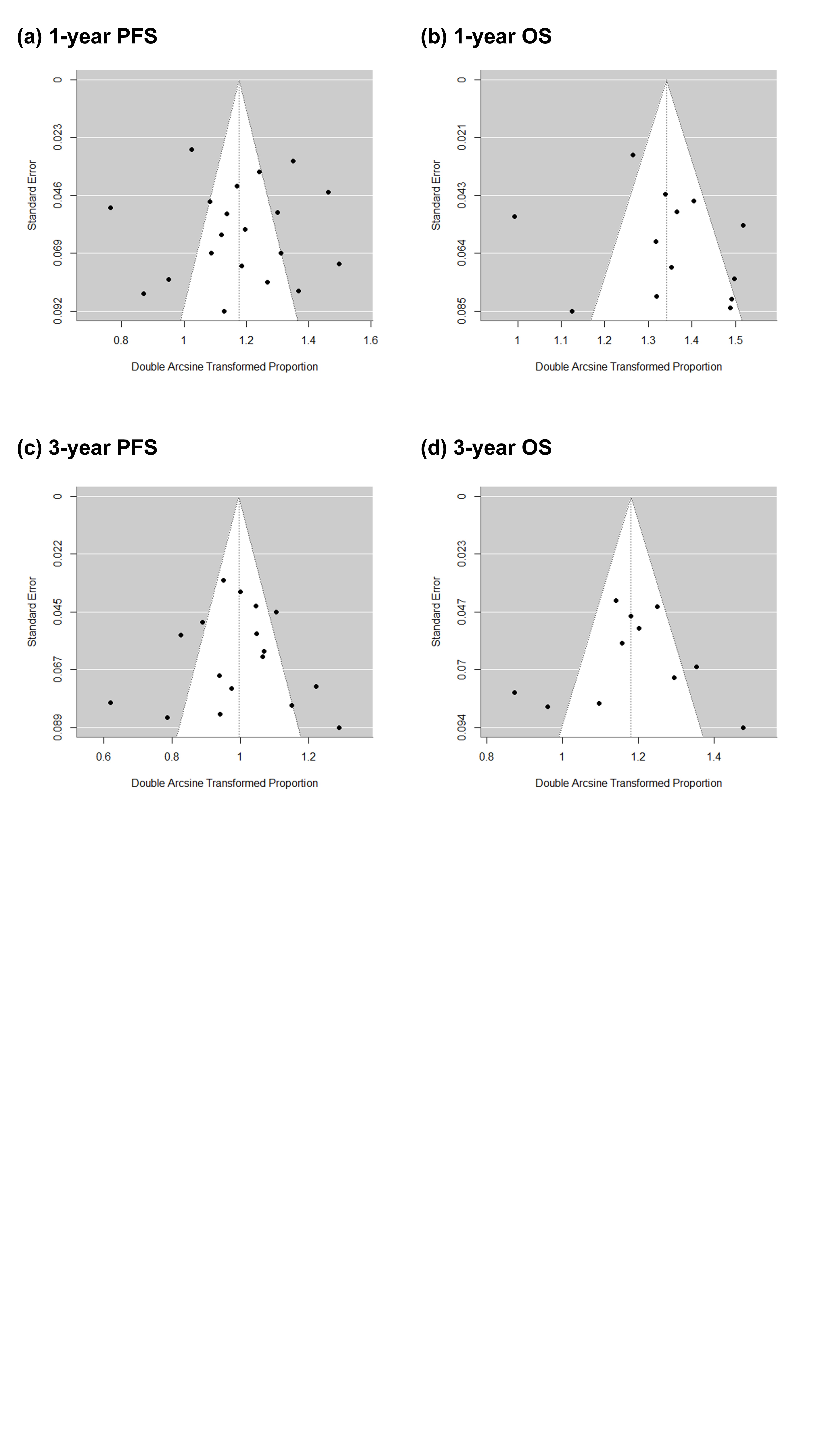

Supplement: Supplementary file 1 — Figure S1. [file JDV-39-1961-s001.jpg]

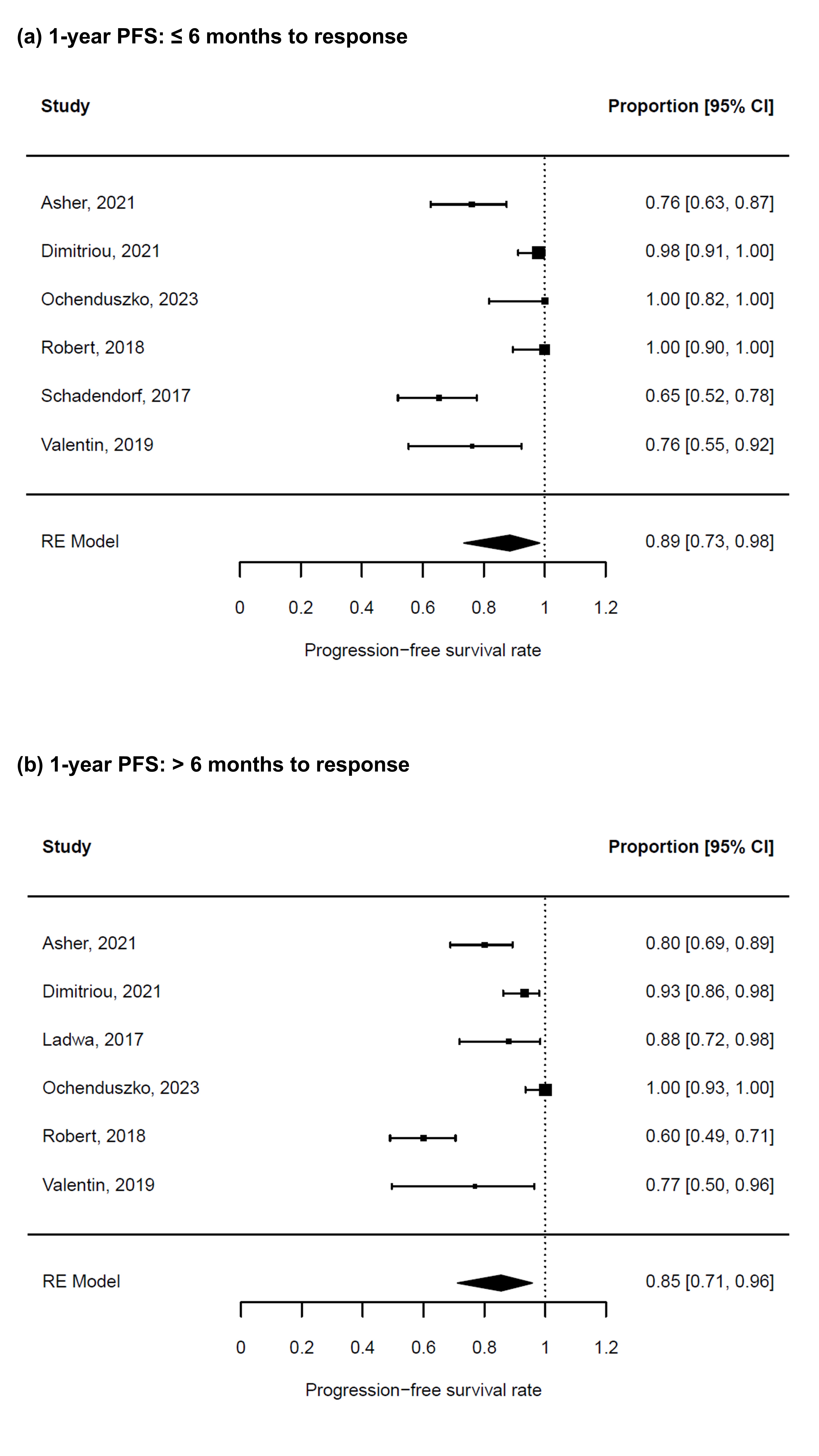

Supplement: Supplementary file 2 — Figure S2. [file JDV-39-1961-s002.jpg]
